# Apoptosis and Inflammation Involved with Fluoride-Induced Bone Injuries

**DOI:** 10.3390/nu16152500

**Published:** 2024-07-31

**Authors:** Miao Wang, Kangting Luo, Tongtong Sha, Qian Li, Zaichao Dong, Yanjie Dou, Huanxia Zhang, Guoyu Zhou, Yue Ba, Fangfang Yu

**Affiliations:** School of Public Health, Zhengzhou University, Zhengzhou 450001, China; wangmiao_9842@163.com (M.W.); lkt1307@163.com (K.L.); shatongtong0802@163.com (T.S.); lqian0103@163.com (Q.L.); dzc0609@163.com (Z.D.); 18835520074@163.com (Y.D.); zhanghuanxia2002@163.com (H.Z.); zhouguoyu@zzu.edu.cn (G.Z.); byyue@zzu.edu.cn (Y.B.)

**Keywords:** fluoride, bone injuries, apoptosis, inflammation

## Abstract

Background: Excessive fluoride exposure induces skeletal fluorosis, but the specific mechanism responsible is still unclear. Therefore, this study aimed to identify the pathogenesis of fluoride-induced bone injuries. Methods: We systematically searched fluoride-induced bone injury-related genes from five databases. Then, these genes were subjected to enrichment analyses. A TF (transcription factor)–mRNA–miRNA network and protein–protein interaction (PPI) network were constructed using Cytoscape, and the Human Protein Atlas (HPA) database was used to screen the expression of key proteins. The candidate pharmacological targets were predicted using the Drug Signature Database. Results: A total of 85 studies were included in this study, and 112 osteoblast-, 35 osteoclast-, and 41 chondrocyte-related differential expression genes (DEGs) were identified. Functional enrichment analyses showed that the Atf4, Bcl2, Col1a1, Fgf21, Fgfr1 and Il6 genes were significantly enriched in the PI3K-Akt signaling pathway of osteoblasts, Mmp9 and Mmp13 genes were enriched in the IL-17 signaling pathway of osteoclasts, and Bmp2 and Bmp7 genes were enriched in the TGF-beta signaling pathway of chondrocytes. With the use of the TF–mRNA–miRNA network, the Col1a1, Bcl2, Fgfr1, Mmp9, Mmp13, Bmp2, and Bmp7 genes were identified as the key regulatory factors. Selenium methyl cysteine, CGS-27023A, and calcium phosphate were predicted to be the potential drugs for skeletal fluorosis. Conclusions: These results suggested that the PI3K-Akt signaling pathway being involved in the apoptosis of osteoblasts, with the IL-17 and the TGF-beta signaling pathways being involved in the inflammation of osteoclasts and chondrocytes in fluoride-induced bone injuries.

## 1. Introduction

Fluorine, an essential trace element for human beings, exists in the environment in the form of fluoride [1]. Fluoride is widely distributed in the soil, rocks, and water throughout the world [2]. Due to volcanic eruptions, mineral dissolution, and human activities, fluoride accumulates in the natural environment [3]. Fluoride has a strong affinity for bones, and a large amount of absorbed fluoride tends to accumulate in bones [4]. Therefore, the concentration of fluoride is closely related to bone development. The World Health Organization (WHO) has established a maximum limit of 1.5 mg/L for fluorine in drinking water [5]. An adequate intake of fluoride promotes the formation of the skeleton [6,7]. On the contrary, excessive accumulation of fluoride can lead to skeletal fluorosis [8,9].

Skeletal fluorosis is a metabolic bone disease, and it mostly involves bone joints. The manifestations of skeletal fluorosis include diffuse osteosclerosis, skeletal pain, connective tissue calcification, and stiffening of bone joints [10,11]. Recent studies have indicated that skeletal fluorosis is associated with the imbalance of bone metabolism, mainly referring to the imbalance between bone formation by osteoblasts and bone resorption by osteoclasts [12,13]. A moderate amount of fluoride promotes the proliferation of osteoblasts and increases bone mass, alkaline phosphatase, bone morphogenetic protein (BMP), and bone gla protein levels [14]. Excessive fluoride leads to the apoptosis of osteoblasts through the activation of caspase-3, resulting in an imbalance of bone remodeling and triggering various bone diseases [15]. Bone resorption in osteoclasts is also essential for normal bone remodeling [16]. Excessive osteoclastic bone resorption can induce osteoporosis and autoimmune arthritis, whereas defective osteoclastic bone resorption can cause osteopetrosis [17]. Moreover, damaged cartilage is common among fluoride-induced bone injuries, primarily including chondrocytes necrosis, proteoglycan changes, a decreased ability of collagen synthesis, and an imbalance of enzyme activity in cartilage tissues [18,19]. Although many more studies have been conducted on the effects of fluoride on osteoblasts, osteoclasts, and chondrocytes, it is still unclear whether fluoride affects bone development via these cells.

Therefore, this study aimed to review studies focusing on fluoride-induced bone injuries and identify fluoride-induced bone injuries-related genes, exploring the pathogenesis of bone disorders’ exposure to fluoride.

## 2. Materials and Methods

### 2.1. Search Strategy

Studies were identified through searching the PubMed, Web of Science, EMBASE, Cochrane Library, and Chinese National Knowledge Infrastructure (CNKI) databases with the keywords (“fluorine” or “fluoride”) and (“skeletal fluorosis” or “SF” or “bone injury”) up to October 2023. The search strategy followed the guidelines of the Preferred Reporting Items for Systematic Reviews and Meta-Analyses (PRISMA) statement.

### 2.2. Inclusion and Exclusion Criteria

We focused on studies that related to fluoride-induced bone injuries. The included studies were selected based on the following criteria: (1) Study design: the rats/mice of fluoride exposure group and control group were included in the study. (2) Study samples: the rats/mice in the fluoride exposure group were exposed to fluoride, while the control group was not exposed to fluoride. (3) Study outcome was associated with fluoride-induced bone injuries.

Studies were excluded based on the following criteria: (1) The full text of the article could not be obtained, contained incomplete data, and/or was unextractable. (2) Was a review, report, and letter. (3) There were duplicate studies from different databases. (4) The outcomes were not associated with fluoride-induced bone injuries.

### 2.3. Study Selection and Data Extraction

The titles and abstracts were reviewed, and the eligibility of selected studies was determined by two investigators (M Wang and FF Yu) independently. After removing studies that obviously violated the inclusion criteria, the remaining articles were reviewed. Any disagreements about study eligibility between the two investigators were resolved through discussion. Subsequently, we systematically searched and identified fluoride-induced bone injury-related genes from the identified studies. These genes were classified into different sets according to the different cell types: osteoblasts, osteoclasts, and chondrocytes.

### 2.4. Gene Ontology and Kyoto Encyclopedia of Genes Genomes Pathway Enrichment Analyses

The Gene Ontology (GO) database (https://www.geneontology.org/, accessed on 15 January 2024) contains information of gene functions. GO analysis annotates genes into biological process (BP), cellular component (CC), and molecular function (MF) terms. The Kyoto Encyclopedia of Genes Genomes (KEGG) database (https://www.genome.jp/kegg/, accessed on 20 January 2024) includes the functional subcategory, route, and annotation of the biological system. In this study, GO and KEGG enrichment analyses were conducted to evaluate the molecular mechanism of fluoride-induced bone injuries, and analysis of the functions of differentially expressed genes (DEGs) was carried out in various cell types using R software v4.3.1 (https://mirrors.tuna.tsinghua.edu.cn/CRAN/, accessed on 1 February 2024).

### 2.5. Protein–Protein Interaction Network Construction

The STRING database (https://cn.string-db.org/, accessed on 6 March 2024) was used to construct the interactive network among the DEGs related to fluoride-induced bone injuries. The interactions were reflected by the comprehensive score, and an interaction score of >0.4 was considered the reliability threshold value [20]. Based on the inter-relationships, the PPI network was established. Then, they were visualized using Cytoscape v3.9.0 (https://cytoscape.org/, accessed on 15 March 2024) [21]. According to Cyto-Hubba, the top five hub genes were screened [22].

### 2.6. Transcription Factor-mRNA-miRNA Regulatory Network Construction and Key Gene Identification

The transcription factors (TFs) and miRNAs of the DEGs that were in the primary pathway were predicted. In this analysis, the TFs were decoded using the GTRD database (https://gtrd20-06.biouml.org, accessed on 20 March 2024), and the miRNAs were identified using the TargetScanMouse database (https://www.targetscan.org/mmu_80/, accessed on 21 March 2024). Furthermore, the interactive network of TF–mRNA–miRNA was constructed via Cytoscape v3.9.0 (https://cytoscape.org/, accessed on 21 March 2024).

The HPA database (https://www.proteinatlas.org/, accessed on 26 March 2024) provides information regarding transcriptome and protein mapping for specific human tissues. In this study, the HPA database was used to obtain the key protein expression levels in skeletal muscle and bone marrow tissues.

### 2.7. Candidate Pharmacological Target Prediction

To identify the candidate drugs that target fluoride-induced bone injuries, we leveraged the Drug Signature Database (DSigDB) via Enrichr v3.2.0 (https://maayanlab.cloud/Enrichr/, accessed on 3 April 2024). The candidate targeted drugs of the key genes were sorted by *p* value, and a *p* value of < 0.05 was considered statistically significant [23].

## 3. Results

### 3.1. General Characteristics

**Figure 1:**
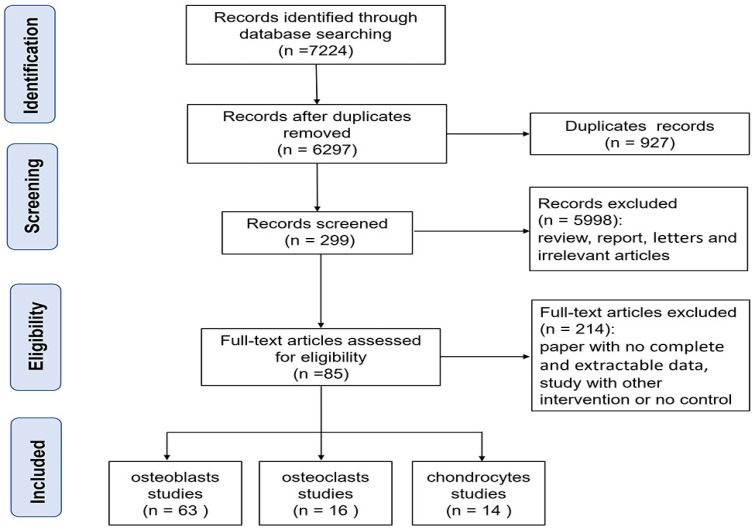
Flow diagram for the study selection procedure.

A total of 7224 relevant articles were identified among the five databases. The selection process of studies is shown in Figure 1. Initially, 927 duplicate records were excluded. Subsequently, the titles and abstracts were reviewed, and 5998 records were excluded after the preliminary screening. Then, full-text articles were evaluated, and 214 records were excluded. Finally, 85 studies were included in this study, with 63 studies focusing on osteoblasts, 16 studies on osteoclasts, and 14 studies on chondrocytes.

### 3.2. Identification and Classification of Differentially Expressed Genes

In accordance with the different cell types (osteoblasts, osteoclasts, and chondrocytes), studies were classified into different groups. The baseline data including 85 studies are presented in Table 1. Among the 63 osteoblast studies, 112 osteoblast-related DEGs were identified between the fluoride exposure group and control group. Among the 16 osteoclast studies, 35 osteoclast-related DEGs were identified between the fluoride exposure group and control group. Among the 14 chondrocyte studies, 41 chondrocyte-related DEGs were identified between the above-mentioned two groups.

### 3.3. Functional Enrichment Analyses of Differentially Expressed Genes

**Figure 2:**
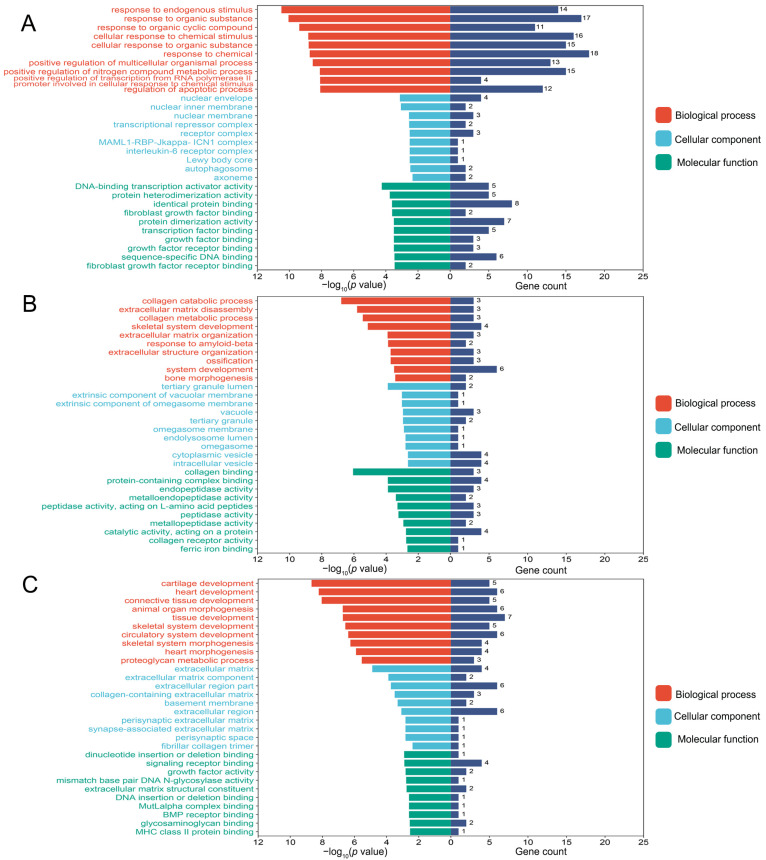
The top 10 biological process (BP), cellular component (CC), and molecular function (MF) terms of the osteoblast-related differentially expressed genes (DEGs) (**A**), osteoclast-related DEGs (**B**), and chondrocyte-related DEGs (**C**) in the fluoride exposure group compared with the control group. *p* value < 0.05 was considered significant.

GO and KEGG enrichment analyses were performed on fluoride-induced bone injuries related to DEGs. As shown in Figure 2A, the top 10 BP, CC, and MF terms were presented according to their *p* value. In osteoblasts, the BP term was significantly enriched in the cellular response to chemical stimulus. The MF term was related to protein heterodimerization activity. In osteoclasts, GO analysis indicated that skeletal system development, ossification, and bone morphogenesis terms were significantly enriched (Figure 2B). In chondrocytes, the BP term was mainly enriched in cartilage development, skeletal system development, and skeletal system morphogenesis. The CC term was enriched in the extracellular matrix, and the MF term was related to signaling receptor binding (Figure 2C).

**Figure 3:**
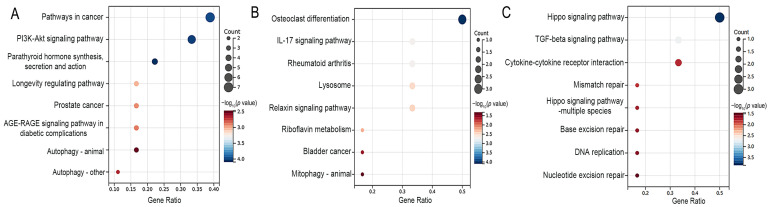
The top 8 enriched signaling pathways of the osteoblast-related DEGs (**A**), osteoclast-related DEGs (**B**), and chondrocyte-related DEGs (**C**) in the fluoride exposure group compared with the control group. Pathways with *p* value < 0.05 were considered significant.

Moreover, KEGG pathway enrichment analysis revealed that Atf4, Bcl2, Col1a1, Fgf21, Fgfr1, and Il6 genes were significantly enriched in the PI3K-Akt signaling pathway of osteoblasts (Figure 3A). Similarly, the Mmp13 and Mmp9 genes were significantly enriched in the IL-17 signaling pathway of osteoclasts (Figure 3B). The Bmp2 and Bmp7 genes were enriched in the TGF-beta signaling pathway of chondrocytes (Figure 3C).

### 3.4. Protein–Protein Interaction Network Analysis

**Figure 4:**
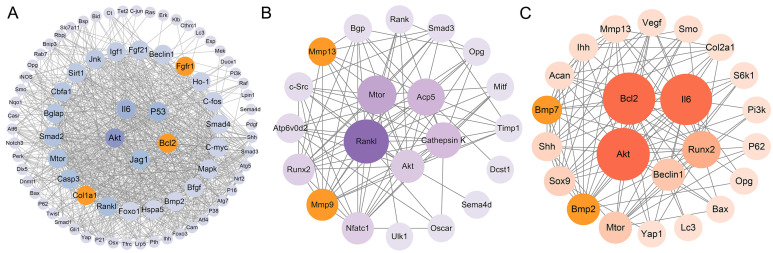
Protein–protein interaction (PPI) network among the osteoblast-related DEGs (**A**), osteoclast-related DEGs (**B**), and chondrocyte-related DEGs (**C**) was constructed by STRING database and visualized by Cytoscape. The top 5 hub genes were identified via the MCC algorithm of the cyto-hubba plug-in. Orange represents the key genes.

The identified DEGs were used to construct the interaction network with STRING, and the results were visualized using Cytoscape. As shown in Figure 4A, the network consisted of 81 nodes and 870 edges, and the top 5 hub genes were Akt, Il6, P53, Bcl2, and Jag1 in the osteoblasts. A total of 21 nodes and 86 edges were shown in the interaction network of osteoclasts, and the Rankl, Mtor, Acp5, Cathepsin K, and Akt genes were identified as the hub genes (Figure 4B). Similarly, 23 nodes and 111 edges were identified in the interaction network of chondrocytes, and the Akt, Bcl2, Il6, Runx2, and Beclin1 genes were ranked as the top 5 hub genes according to the Maximal Clique Centrality (MCC) algorithm (Figure 4C).

### 3.5. Transcription Factor–mRNA–miRNA Regulatory Network Construction and Key Gene Identification

**Figure 5:**
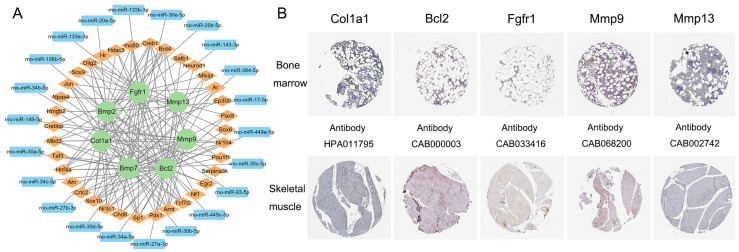
The transcription factor (TF)–mRNA–miRNA regulatory network (**A**) and immunohistochemistry images of the key genes from the HPA database (**B**). Green represents the key genes. Yellow represents the target TFs. Blue represents the target miRNAs. The scale bar is 200 µm.

According to the TargetScanMouse database, the Atf4, Fgf21, and Il6 genes were not predicted to target any miRNAs, so they were excluded from the key genes. Combined with the GTRD database, we found that a total of 35 TFs and 22 miRNAs regulated the expression of key genes. Eventually, 131 TF–mRNA pairs and 22 miRNA–mRNA pairs were integrated to construct a TF–miRNA–mRNA regulatory network (Figure 5A).

As shown in Figure 5B, the key genes of Col1a1, Bcl2, Fgfr1, Mmp9, and Mmp13 were screened using the HPA database. These genes were expressed both in bone marrow and skeletal muscle tissues. Unfortunately, the normal tissue analysis of Bmp2 and Bmp7 required more information.

### 3.6. Prediction of Candidate Drugs

The pharmacological targets of fluoride-induced bone injuries were predicted via the Enrichr platform. The top 5 candidate drugs were listed according to their *p* value (Table 2). These results showed that selenium methyl cysteine (CTD 00000103), CGS-27023A (TTD 00002801) and calcium phosphate (BOSS) were possibly the key drugs responsible for skeletal fluorosis.

## 4. Discussion

**Figure 6:**
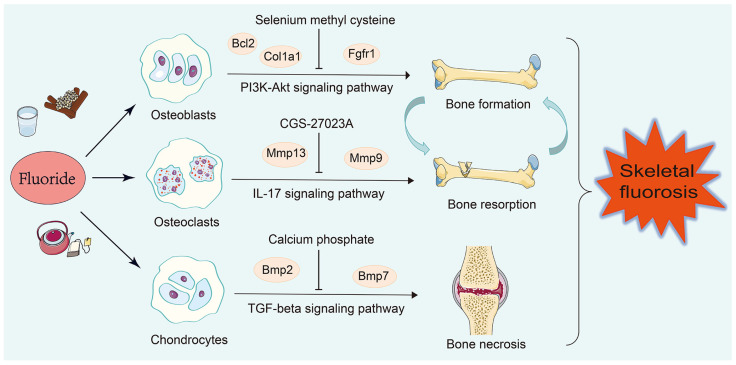
The regulatory mechanism of bone injuries induced by fluoride. The Bcl2, Col1a1, and Fgfr1 genes were involved in the apoptosis of osteoblasts via the PI3K-Akt signaling pathway, inhibiting bone formation. The Mmp13 and Mmp9 genes caused the inflammation of osteoclasts by activating the IL-17 signaling pathway, promoting bone resorption. The Bmp2 and Bmp7 genes caused the inflammation of chondrocytes by activating the TGF-beta signaling pathway, resulting in bone necrosis.

Fluoride affects bone metabolism by intervening with osteoblast and osteoclast activity, and fluoride exposure also triggers chondrocyte degradation [109,110]. To shed light on the specific mechanisms of fluoride-induced bone injuries, we discovered that the apoptosis of osteoblasts and the inflammation of osteoclasts and chondrocytes were involved in fluoride-induced bone injuries. Additionally, the Col1a1, Bcl2, Fgfr1, Mmp9, Mmp13, Bmp2, and Bmp7 genes were identified as the key regulatory factors of fluoride-induced bone injuries (Figure 6).

In this study, a total of 112, 35, and 41 DEGs were obtained in the identified 63 osteoblast studies, 16 osteoclast studies, and 14 chondrocyte studies, respectively. According to the KEGG enrichment analysis in osteoblasts, the PI3K-Akt signaling pathway was significantly enriched. The PI3K-Akt signaling pathway was closely associated with bone metabolism [111,112,113]. Studies demonstrated that the PI3K-Akt signaling pathway participated in the excessive proliferation and differentiation of osteoblasts in rats [86]. Combined with the TF–mRNA–miRNA regulatory network, the Col1a1, Bcl2, and Fgfr1 genes were identified as the key genes for bone formation, which was regulated by the PI3K-Akt signaling pathway (Figure S1). Col1a1 is a hydrophilic protein that belongs to the collagen family and is closely associated with osteogenesis imperfecta, osteoporosis, and other skeletal injuries [114,115,116]. The LINC00313/miR-218-5p/COL1A1 axis contributed to osimertinib resistance through the PI3K-Akt signaling pathway, confirming that Col1a1 is likely to be the upstream gene for the PI3K-Akt signaling pathway [117]. Additionally, the overexpression of Fgfr1 promoted the activation of the PI3K-Akt pathway [118]. Fgfr1 plays essential roles in osteocytes during bone remodeling, and this gene is suggested to be a potential therapeutic target for the prevention of bone loss [119]. Moreover, the low expression of Akt increased the level of the pro-apoptotic protein Bax, which failed to form heterodimers with the anti-apoptotic protein Bcl2, resulting in osteoblast apoptosis [120]. Based on these studies, fluoride may activate Col1a1, Bcl2, and Fgfr1 and regulate osteoblast apoptosis through the PI3K-Akt signaling pathway. Furthermore, selenium methyl cysteine (CTD 00000103) was predicted to be a potential drug according to the DSigDB database. Supplementation with selenium methyl cysteine increased bone mineral density [121]. Studies have demonstrated that selenium methyl cysteine protects against liver injuries by inhibiting apoptosis [122]. In the future, it could be applied for treating fluoride-induced bone injuries based on these findings.

Osteoclasts are a type of multinucleated bone-resorbing cells that originate from the myeloid lineage of hematopoietic stem cells in bone marrow [123]. In our study, GO analysis indicated that osteoclasts were responsible for bone resorption. Moreover, the Mmp13 and Mmp9 genes were mainly enriched in the IL17 signaling pathway (Figure S2). IL-17 is a proinflammatory cytokine. Recent studies have shown that IL-17 promotes osteoclast-induced bone loss by regulating glutamine-dependent energy metabolism. Moreover, IL-17 treatment increases the expression of osteoclast marker genes Mmp9 and Mmp13 [124]. The Mmp9 and Mmp13 genes are involved in the process of bone resorption through the activation and differentiation of osteoclasts [125,126]. In this study, we found that fluoride exposure activated the Mmp9 and Mmp13 genes and regulated bone resorption through the IL-17 signaling pathway. Among the top 5 candidate drugs in osteoclasts, CGS-27023A was identified as a potent matrix metalloproteinase inhibitor. It can be utilized to regulate the process of bone resorption through Mmp13 and Mmp9 [127].

Furthermore, we focused on the enrichment analysis of chondrocyte-related DEGs. GO analysis suggested that chondrocytes were significantly important for the development of the skeletal system. KEGG enrichment analysis showed that the Bmp2 and Bmp7 genes were mainly enriched in the TGF-beta signaling pathway (Figure S3). BMPs are a type of extracellular multifunctional signaling cytokine that belongs to the TGF-beta family [128]. Bmp2 and Bmp7 have been shown to induce endochondral bone formation and subsequently form long bones [129,130]. The high expression of Bmp2, along with TGF-beta1, promotes the expansion of Treg cells, leading to significant inflammation [7,131]. A large number of studies has also confirmed that fluoride exposure leads to an inflammatory response [132,133,134]. Moreover, the PPI analysis identified several hub proteins related to inflammation, such as Akt, Il6, Runx2, Bcl2, and Beclin1 [135,136,137,138]. Studies have shown that TGF-beta degrades the extracellular matrix, induces chondrocyte differentiation, and even leads to bone necrosis [139,140]. According to the analysis of candidate drugs, calcium phosphate is supposed to be used for inhibiting bone necrosis. Studies have indicated that calcium phosphate could be used in bone grafts due to its composition, supporting our findings [141].

Overall, the above findings provide promising insights for skeletal fluorosis. However, our study still has certain limitations. The specific regulatory mechanisms of the PI3K-Akt signaling pathway, IL-17 signaling pathway, and TGF-beta signaling pathway in bone disorders’ exposure to fluoride are needed to be further verified via in vivo and in vitro experiments.

## 5. Conclusions

In conclusion, the PI3K-Akt signaling pathway is involved in the apoptosis of osteoblasts and the IL-17 and TGF-beta signaling pathways are involved in the inflammation of osteoclasts and chondrocytes, playing a part in the process of fluoride-induced bone injuries. The Col1a1, Bcl2, Fgfr1, Mmp9, Mmp13, Bmp2, and Bmp7 genes are the key regulatory factors in fluorosis bone metabolism.

## Figures and Tables

**Table 1:** Basic information of included studies.

Cell Type	Literature Number	Genes	Gene Number
Osteoblasts	63 [24,25,26,27,28,29,30,31,32,33,34,35,36,37,38,39,40,41,42,43,44,45,46,47,48,49,50,51,52,53,54,55,56,57,58,59,60,61,62,63,64,65,66,67,68,69,70,71,72,73,74,75,76,77,78,79,80,81,82,83,84,85,86]	Acetyl-p53, Acsl3, Akt, Akt1, Aloxe3, Alp, Atf4, Atf6, Atg5, Atg7, Bmp2, Bax, Bcl2, Beclin 1, Bfgf, Bgp, Bid, Bip, Bnip3, Bsp, CaM, CaN, Caspase-3, Casr, Cbfa1, Cdo1, c-fos, c-jun, C-myc, Col, Col I, Col1a1, Ct, Cthrc1, CyclinD1, Cyt C, Dlx5, Dnmt1, Duox1, Dv1, Enpp2, Erk, Esp, Fgf21, Fgfr1, Foxo1, Foxo3, Gli1, Gsk3β, Gsto1, Ho-1, Igf1, Ihh, Il6, iNOS, Insr, Ire1, Jag1, Jnk, Klb, Lc3, Lpin1, Lrp, Lrp5, Mapk, Mcm3, Mek, Mtor, Notch3, Nqo1, Nrf2, Ocn, Opg, Opn, Osx, p16, p21, p38, p53, p62, Pdgf, Perk, Pi3k, Pth, Pth-rp, Rab7, Raf, Rankl, Ras, Rbpj, Runx2, Sema4d, Shh, Sirt1, Slc7a11, Smad1, Smad2, Smad3, Smad4, Smo, Tet2, Tfrc, Tgf-β1, Tgf-β, Tm9sf1, Twist, Tβr2, Wnt10, Wnt3α, Xbp-1, Yap, β-catenin	112
Osteoclasts	16 [44,56,59,62,72,74,83,87,88,89,90,91,92,93,94,95]	β-catenin, Acp5, Akt, Alp, Atp6v0d2, Bgp, Cathepsin K, Col1α1, c-Src, Ctsk, Dcst1, Erα, Ifnγ, Mitf, Mmp13, Mmp9, Mtor, Nfatc1, Nf-κb1, Opg, Oscar, Osterix, Rank, Rankl, Rcthrc1, Runx2, Sema4d, Smad3, Tgf-β1, Timp-1, Tracp, Traf-6, Trap, Tβr1, Ulk1	35
Chondrocytes	14 [44,96,97,98,99,100,101,102,103,104,105,106,107,108]	4Ebp1, Acan, Aggrecan, Akt, Bax, Bcl2, Beclin1, Bmp2, Bmp7, Caspase14, Cleaved caspase 12, Cleaved caspase 3, Cleaved caspase 9, Col II, Col X, Col2a1, Colixa3, Hif-1α, Ihh, Il6, Lc3, Mig-6, Mmp13, Mtor, Opg, P62, Pcna, P-egfr, Pi3k, P-lats1/2, P-mst1/2, Ps6, P-thrp, Runx2, S6k1, Shh, Smo, Sox9, Vegf, Yap1, β-catenin	41

**Table 2:** Prediction of the top 5 candidate drugs in three cell types.

Cell Type	Candidate Drugs
Osteoblasts	Pamidronate (CTD 00000975), Geranylgeranyl pyrophosphate (CTD 00000102), Selenium methyl cysteine (CTD 00000103), Cyclohexanecarboxamide (CTD 00003513), Paricalcitol (CTD 00003033)
Osteoclasts	Ilomastat (TTD 00008545), CGS-27023A (TTD 00002801), CHEMBL475540 (TTD 00006054), doxycycline (CTD 00005875), Toluidine Blue O (BOSS)
Chondrocytes	Octreotide (CTD 00007059), Stannic fluoride (BOSS), Calcium phosphate (BOSS), Heparitin (BOSS), TITANIUM (BOSS)

## Data Availability

Data is contained within the article.

## Supplementary Materials

The following supporting information can be downloaded at: https://www.mdpi.com/article/10.3390/nu16152500/s1, Figure S1: The Fgfr1, Bcl2, and Col1a1 genes involved in the PI3K-Akt signaling pathway; Figure S2: The Mmp9 and Mmp13 genes involved in the IL-17 signaling pathway; Figure S3: The Bmp2 and Bmp7 genes involved in the TGF-beta signaling pathway. The positions of key genes are highlighted.
