# Supplementary figures and images for: Apoptosis and Inflammation Involved with Fluoride-Induced Bone Injuries
Source: Nutrients. 2024 Jul 31;16(15):2500. doi: 10.3390/nu16152500 (PMC11313706; doi:10.3390/nu16152500)

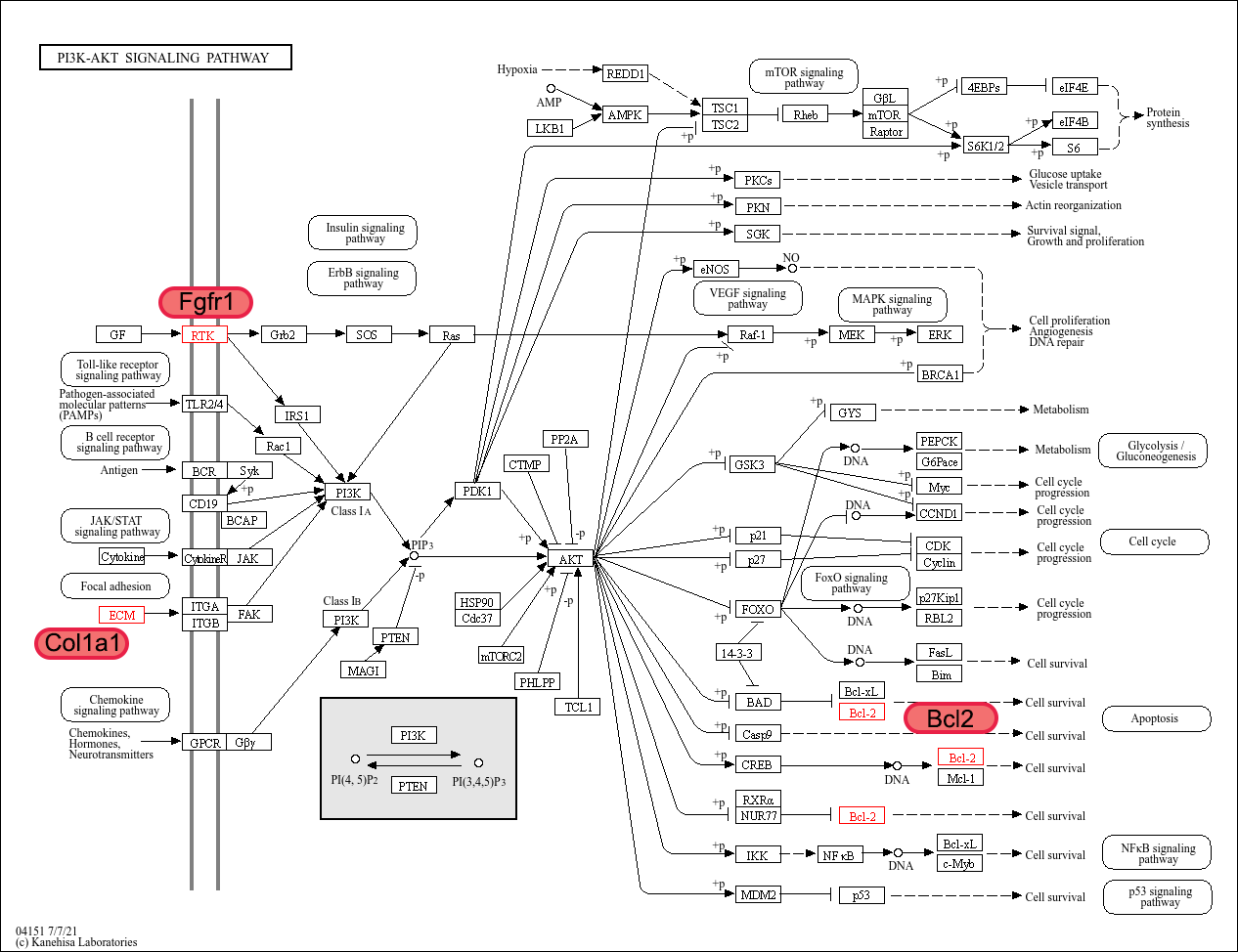

Supplement: Supplementary file 1 [file nutrients-16-02500-s001.zip › Supplementary Files/Figure S1.tif]

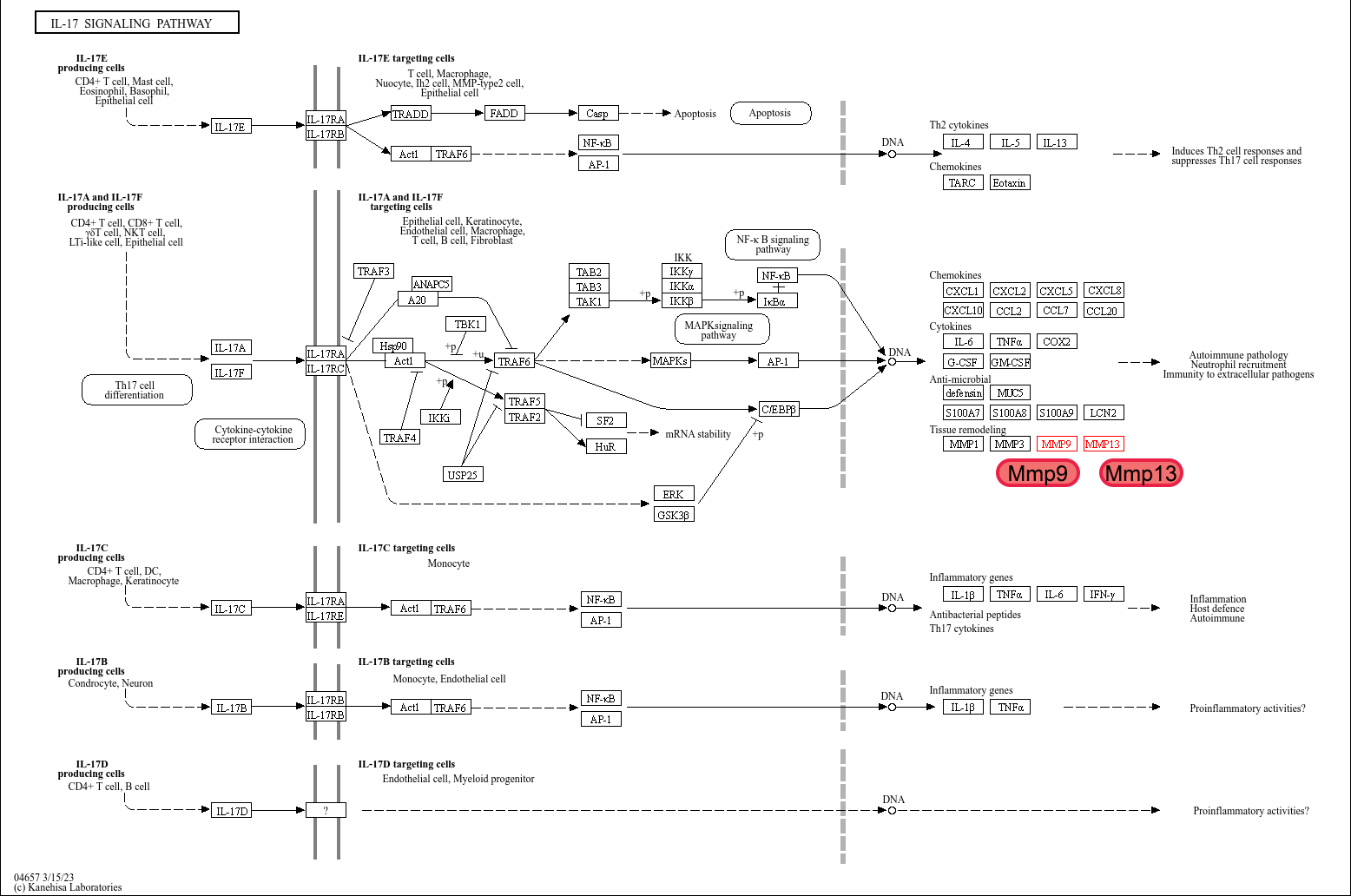

Supplement: Supplementary file 1 [file nutrients-16-02500-s001.zip › Supplementary Files/Figure S2.tif]

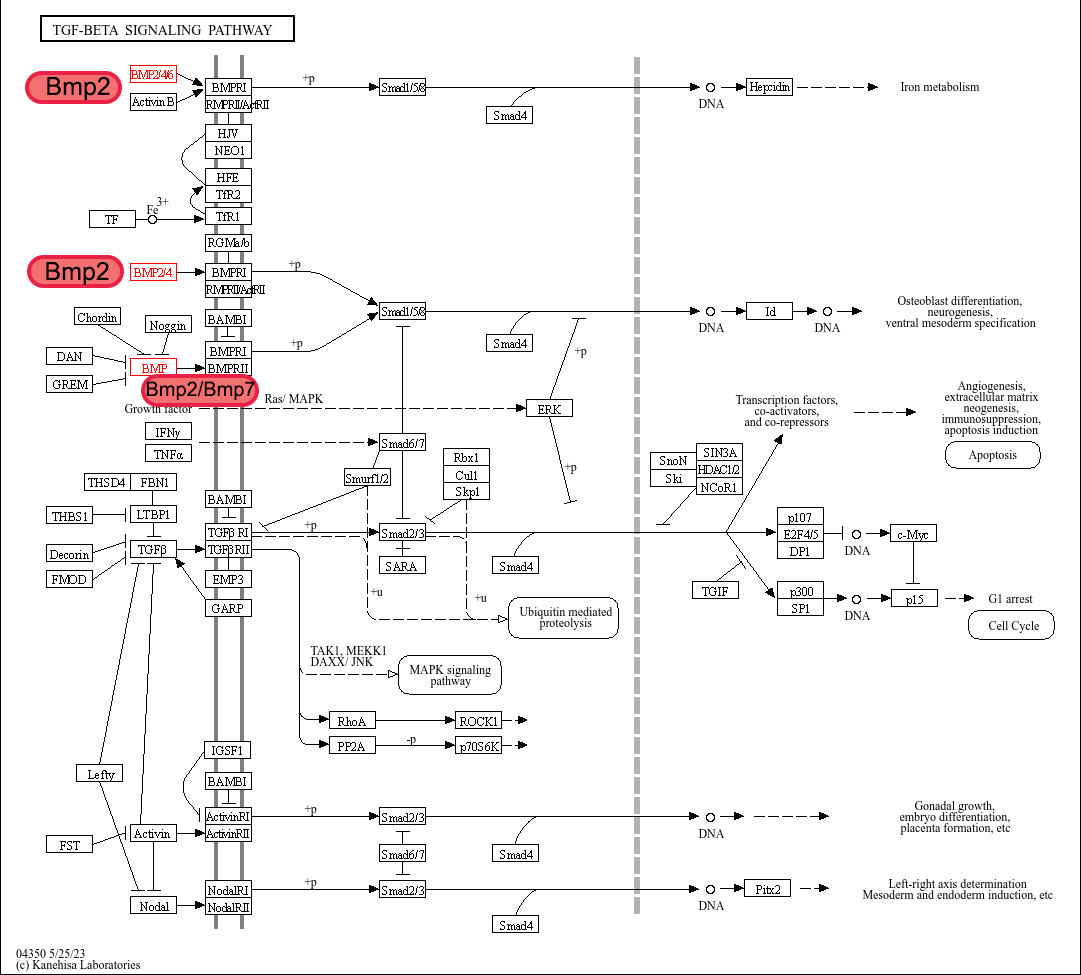

Supplement: Supplementary file 1 [file nutrients-16-02500-s001.zip › Supplementary Files/Figure S3.tif]
